# Studying Consumer Behavior in an Online Context: The Impact of the Evolution of the World Wide Web for New Avenues in Research

**DOI:** 10.3389/fpsyg.2019.02731

**Published:** 2019-12-03

**Authors:** Maria Pilar Martinez-Ruiz, Karin S. Moser

**Affiliations:** ^1^Department of Business Administration, University of Castilla La Mancha, Albacete, Spain; ^2^Business School, London South Bank University, London, United Kingdom

**Keywords:** consumer behavior, online context, new research agenda, World Wide Web evolution, Web 5.0, decision making, sensory experience, emotions

## Introduction

There is no denying that the increasing use of the internet by end consumers has presented numerous challenges in the field of marketing research, and more specifically in the field of consumer behavior (Pomirleanu et al., 2013) as evidenced by a growing number of studies (Cummins et al., 2014).

Understanding the psychology behind online consumer behavior is key to compete in today's markets which are characterized by ever increasing competition and globalization. In an online context, consumer responses are no longer dependent on the physical environment while at the same time entirely new factors come into play such as the device through which consumers interact, and the way products and services are sold and presented online which often differs significantly from traditional offline marketing strategies. It is for this reason that research into online consumer behavior has increasingly started looking to other disciplines, including psychological approaches and concepts.

Several examples of cornerstone reviews highlight this trend. For example, Cummins et al. (2014) observed in their literature review how research on online consumer behavior often uses psychological and social networks theories. The authors found a much greater focus on aspects related to the relationships that consumers develop with companies over the internet and connected to that, the analysis of the attitudes, beliefs and feelings that consumers develop as part of their online experiences, thus moving the relationship from the physical into the virtual sphere. Similarly, Yadav and Pavlou (2014) did a review of marketing literature in computer-mediated environments (CME) and proposed a structured framework around four key interactions in CME to summarize their findings: consumer-firm interactions, firm-consumer interactions, consumer-consumer interactions, and firm-firm interactions. Both reviews support our suggestion that understanding consumer behavior in a digital world relates to important psychological aspects of behavior that are best understood by using psychological concepts and methods.

Despite this evidence there are surprisingly still some important avenues of research that have received little attention. One area that we propose is central to understanding online consumer behavior concerns the impact of the different stages of internet evolution, the World Wide Web (WWW). This has received some attention in general management research and from practitioners but there is hardly any research in marketing and consumer behavior. The WWW is omnipresent in business today, and almost all work-related communications and interactions are at least partially supported by digital media (Moser and Axtell, 2013). It has transformed business operations and relations (Benito-Osorio et al., 2013; Kambil, 2008) and it is time to extend this to customer relations and marketing.

It is the aim of this article to highlight the importance of studying the impact of the evolution of the WWW on consumer behavior. Especially in the most recent WWW developments, psychological aspects of consumer behavior have gained in importance, namely individual preferences, emotions, and sensory experiences. In order to analyse these transformations, it is important to briefly consider what each stage in the evolution of the WWW has brought about.

## The Evolution of the World Wide Web and Its Impact on Consumer Behavior

The first stage in the evolution of the WWW was the Web 1.0 (usually referred to as the Basic Web). This type of WWW was suitable mainly for sharing information on a new platform, such as publishing corporate information online and conducting basic business transactions. It was the first opportunity for businesses to develop an online presence and was mainly used by large international companies (Berners-Lee et al., 1992; Benito-Osorio et al., 2013). Only a fraction of consumers used it to stay informed and so its reach was restricted to the early adopters of the new technology. Its limited reach and suitability for large companies and basic tasks only meant that the Web 1.0 was mainly used for transactional marketing. Web 1.0 already showed the potential for online relationship marketing (for example, the possibility for consumers to access a greater breadth of information about products and brands than was physically available on the ground). Mostly, online consumer behavior was still seen as only complementary to traditional consumer behavior. Just a few pioneering publications highlighted the enormous potential of the WWW as information and communication channel that could completely reconfigure the ways in which consumers seek information and compare knowledge about products and services (Choo et al., 2000).

The Web 2.0 (known as the Social Web) was a big step forward by evolving into a platform for collaboration. The WWW was no longer a repository of information but an enabler of social interaction and collaboration on a global scale, with potentially anyone across the globe who had internet access. Hence, the Web 2.0 was qualitatively completely different from the previous Web 1.0 technologies as it began to facilitate information sharing between users, employed user-centered design technology, and supported interoperability and collaboration. This also meant that the online platforms were now open to a much wider range of users, not just the large multinationals with their own IT departments, but also to SMEs, independents, and individual consumers. Typical examples of the Social Web are Web-based communities, social network services, video hosting services, wikis, and blogs (Benito-Osorio et al., 2013). The internet could now be used to organize individuals for collective action, initiate trends, promote views, and take influence. This opened up the possibility of generating extraordinary value by creating new movements and communities that hadn't existed before and that could only be reached via the new digital technologies. The Web 2.0 fostered the building of bidirectional relations between consumers and providers of goods and services. With this, we posit that the WWW entered the next stage as the key medium for relationship marketing which in turn became central for business success.

The third step in the evolution of the WWW was the Web 3.0, the Semantic Web. Although there is still some debate over the significance and most appropriate definition of the Web 3.0, it is indisputable that one of its key features was the combination of human and artificial intelligence. This allowed to provide more relevant and more easily accessible information that was specifically targeted to groups of consumers and based on their real time online behavior thanks to non-browser-based applications and AI technologies (Benito-Osorio et al., 2013). It further increased the amount and breadth of available information, in real time and in an interactive way that took into account which decisions consumers made when browsing online and responding to prompts and targeted information. Companies and internet service providers could now gather detailed information about consumers' online decision making, with the possibility to be highly adaptive and respond to consumer decisions by offering products and services, for example sales offers or comparisons with similar products in real time. From a research perspective, it was now possible to obtain detailed information about consumer decision making for psychological variables that previously were almost impossible to study in real time: individual perceptions, judgements, attitudes, and intentions toward products, services and people could now be observed at each step in the decision process.

The fourth step in WWW evolution was the development of the Web 4.0 based on wireless communication and mobile devices. The Web 4.0 is usually referred to as the Symbiotic Web, because of its capability to connect people, places and objects whenever and wherever they might be, both in the physical and the virtual world, and in real time. For example, the GPS systems that guide cars and help drivers to improve route planning will in the next step save them from having to drive at all with the introduction of self-driving vehicles. The Web 4.0 is where we currently stand, with its next steps ready to take off and prototypes such as the self-driving vehicles soon ready for mass production. However, we would like to propose that for consumer behavior research we should not stop here but already look to the next evolutionary stage of the WWW, the Web 5.0.

## The Future of Consumption With the Web 5.0

This next step in web evolution is already hard on the heels of the Web 4.0 (Kambil, 2008), with the Web 5.0 currently being in development and referred to as the Sensory Web or the Emotive Web. With these latest and most current developments comes another exponential increase in web-based interactions via multiple channels with responses in real time and adaptive technologies that are immediately predicting and shaping the next interaction. This further evolution of the WWW marks a considerable qualitative difference compared to the Web 4.0 similar in significance to the milestone of moving from the Basic Web 1.0 to the Social Web 2.0 two decades ago. This is in line with the Marketing 4.0, which emphasized the relevance of individual customer experiences and customer journeys for consumer behavior and decision making, adding entirely new aspects to consumer research (Lemon and Verhoef, 2016; Kotler et al., 2017).

Various futuristic terms are currently in use for the Web 5.0, the Sensory and Emotive Web. The aim is to develop computers that interact with human beings and become part of daily consumer and customer relationships for many if not all people. Although the current Web technologies are not yet “emotionally sensitive” or able to read human emotions, these technologies are in development and rapidly advancing. In some very recent studies, computers have already been shown to be able to more accurately judge human emotions and personality attributes from facial expressions in photographs and videos than human observers (Khambatta et al., 2019). A current commercial example already in use is www.wefeelfine.org which tracks emotional phrases on the Web, categorizes them and registers the frequency and location of clusters of expressed sentiments. Another example is the company EmotiveSystems that developed a new neurotechnology using headphones where users can interact with content that responds to their emotions and changes the facial expression of their avatars in real time. These new technologies of the future Web 5.0 will undoubtedly and radically change the way we study and understand consumer behavior.

## Discussion

This short account of the evolution of the WWW shows the multifaceted and massive implications and possibilities for the field of consumer behavior research. We strongly believe that this research is still in its beginning, and that greater efforts should be devoted to research that takes the WWW evolution into account for different sectors, products, services, people, and the increasing importance of consumer perceptions and emotions, and thus inherently psychological aspects of behavior.

Examples are emotions that consumers experience when shopping online and the micro-interactions in consumer decision-making when each choice in the purchase decision process is immediately followed by an individualized response based on AI algorithms, such as offering additional color or size options, which in turn then influence the consumer perception and their next decision and so on. With the further development of the Web 5.0 this will not only be the case for purchasing decisions which are mainly based on cognitive information processing but increasingly also for emotions that consumers experience and express while shopping online, many of which they may not be conscious of. The new emotionally sensitive AI technologies can read (unconscious) emotions based on verbal and facial expressions (from text, speech, photographic, and video data) but in future possibly also based on body sensors measuring heart rate, skin resistance, bodily secretions (sweat), and pupil dilatation. These are established measures in psychological research studying emotional and physiological arousal and this type of data could be obtained via cameras, smart glasses and contact lenses and smart watches.

This will bring a host of new methods into consumer research that are drawn from psychology and can only be developed in an interdisciplinary approach. Given that any decision-making process is highly complex and given that the role of emotions and sensory experiences become ever more prominent with the Web 4.0 and the forthcoming Web 5.0, it is important to start developing adequate methods and theoretical models to study these new developments in consumer behavior.

## Author Contributions

All authors listed have made a substantial, direct and intellectual contribution to the work, and approved it for publication.

### Conflict of Interest

The authors declare that the research was conducted in the absence of any commercial or financial relationships that could be construed as a potential conflict of interest.
